# 

**Published:** 1907-08-15

**Authors:** 


### ANNOUNCEMENTS.

LEBANON VALLEY DENTAL ASSOCIATION.—At the thirty-second annual meeting of the Association officers for the ensuing year were elected as follows: President, Dr. G. S. Schlegel, Reading; vice-president, William B. Mausteller, Harrisburg; recording secretary, H. J. Herbein, Pottsville; corresponding secretary, P. K. Filhert, Pottsville, and treasurer, C. B. Wagner, Lebanon. The executive committee was appointed as follows: H. Elmer Trostle, York, chairman; C. E. Grim, Reading, and H. W. Bohn, Reading. York was selected as the meeting place of next year, and the meeting will take place the second Tuesday in May, instead of the third.—*Harrisburg Telegraph.*

THE ILLINOIS STATE DENTAL SOCIETY.—This society in session at Quincy, May 17, 1907, elected the following officers: W. H. Johnson of Peoria, president; H. L. Whipple, Quincy, vice-president; Arthur D. Black, Chicago, secretary; C. P. Pruyn, Chicago treasurer; J. T. Cummins, Metropolis, librarian; E. K. Blaire of Waverly, W. H. G. Logan of Chicago, and B. Newsom of Minonk, new members council; E. L. Evans of Decatur, and F. W. Gethro of Chicago, clinics committee. Springfield was chosen as the next place of meeting.

THE WEST VIRGINIA STATE DENTAL SOCIETY.—This society organized at Clarksburg, May 21st, with thirty-four charter members. The first regular meeting will be held at Parkersburg, W. Va., the Second Wednesday in October, lasting three days. The following officers were elected: President, H. H. Harrison, Wheeling; vice-president, Chas. H. Bartlett, Parkersburg.

KENTUCKY STATE DENTAL ASSOCIATION.—At the thirty-seventh annual meeting of the Association at Louisville the following officers were elected for the coming year: President, Dr. McFerran Crow, of Versailles; vice-President, Dr. I. H. Harrington, of Louisville; secretary, Dr. W. M. Randall, of Louisville; treasurer, Dr. H. K. Kellogg, of Louisville. Drs. Randall and Kellogg were re-elected to their respective offices. Dr. J. Richard Wallace, of Louisville, was re-elected president ot the State Board of Dental Examiners, and Dr. J. R. Wilder, of Louisville, was elected a member of the Board of Censors, to succeed Raymond E. Grant, whose term of office expired.

---

THE NATIONAL DENTAL ASSOCIATION'S OFFICERS 1907-08.—The National Dental Association at its 11th annuanl session, Minneapolis, July 31st., elected the following officers for the ensuing year: Pres. Wm. Carr, New York City; Vice Pres. for the East, Wilbur F. Litch, Philadelphia, Pa.; Vice President for the South, J. P. Gray, Nashville, Tenn.; Vice President for the West, Alfred Owre, Minneapolis, Minn; Cor. Secy., Burton Lee Thorpe, St. Louis, Mo.; Rec. Secy. Chas. S. Butler, Buffalo, N. Y.; Treas. A. S. Melendy, Knoxville, Tenn. Executive Committee. New Members. L. Meisenburger, Buffalo, N. Y.; F. B. Kremer, Minneapolis, Minn.; M. F. Finley, Washington, D. C. Executive Council. H. J. Burkhart, chairman, Batavia, N. Y; J. Y. Crawtord, Nashville, Tenn.; A. H. Peck, Chicago, Ills.; F. O. Hetrick, Ottawa, Kansas.; B. Holly Smith, Baltimore, Md. Next place of meeting, Boston, 1908.

---

REVISED PROGRAM FOR THE JAMESTOWN CONVENTION MEETING to be held in the Convention Hall, exposition grounds, Norfolk, Va., Sept. 10, 11, 12, 1907.

Tuesday, Sept. 10, 1907. 9:30 A. M. Meeting called to order by Dr. Burton Lee Thorpe, St. Louis, Chairman Committee on Organization. Invocation, Rev. C. L. Bane. Pastor Memorial M. E. Church, Norfolk, Va. Address of welcome, Harry St. George Tucker, President Jamestown Exposition Co. Address of welcome, Dr. Edward Eggleston,

Richmond, President Virginia State Dental Association. Address of welcome, Dr. Joseph W. Eggleston, Richmond, Va., in behalf of the profession of Virginia; Address of welcome, Dr. W. G. Mason, Tampa, Fla., President Southern Branch N. D. A., Address of welcome, Dr. J. Y. Crawford, Nashville, Tenn., in behalf of the profession of the South; Response to the address of welcome, Dr. J. D. Patterson, Kansas City, Mo.; Address by the President, Dr. V. E. Turner, Raleigh, N. C.

Tuesday afternoon session 2:30 P. M. Clinics in Convention Hall, Dr. Clarence J. Grieves, Chairman, Baltimore, Md. Tuesday evening, Sept. 10, 8:00 P. M. Smoker at Inside Inn, Dr. B. Holly Smith, Chairman, Baltimore, Md.

Wednesday morning, Sept. 11, 9:30 A. M. Illustrated Lecture, Dr. F. T. Van Woert, Brooklyn, N. Y. "Is the Cemented Filling, the Filling of the Future?"; Discussion opened by Dr. Wm. K. Slater, Knoxville, Tenn., and Dr. Craig M. Work, Ottumwa, Iowa. 11:00 A. M. Illustrated lecture, Dr. Chas. L. Alexander, Charlotte, N. C. "Gold Inlays."; Discussion opened by Dr. Herbert Johnson, Macon, Ga., and Dr. J. G. Fife, Dallas, Texas. 2:30 P.M. Clinics in Convention Hall. 8:00 P. M. Illustrated lecture, Dr. R. Ottolengui, New York City, "The Purposes and Accomplishments of Modern Orthodontia"; Discussion opened by Dr. W. C. Talbot, New Orleans, La., and Dr. Henry W. Morgan, Nashville, Tenn.

Thursday, 9:30 A. M. Clinics in Convention Hall 2:30 P. M. Special Clinical Lecture and Demonstration by Dr. Wm. H. Taggart, Chicago, "Cast Gold Inlays, Bridges and Plates."; Discussion opened by Dr. J. H. Lorenz, Atlanta, Ga., and Dr. L. E. Custer, Dayton, Ohio. Adjournment. 8:00 P. M. Entertainment given to members and guests of the convention by Virginia State Dental Associaton under the chairmanship of the Society's President, Dr. Edward Eggleston, Richmond, Va.

A cordial invitation is extended to all ethical dentists to become members and attend this meeting.

*All sessions are to be held in "The Convention Hall," at Exposition Grounds. Entrance to this hall is outside of the grounds. Thus saving admission fee to enter it, however entrance to the grounds is possible without leaving the hall.*

To expidite the work before the general sessions all. resolutions, motions and routine business must first be submitted to the Committee on Organization who at the proper time will present it to the general body.

